# Dengue fever in the Americas

**DOI:** 10.1080/21505594.2024.2375551

**Published:** 2024-07-11

**Authors:** Hinh Ly

**Affiliations:** Department of Veterinary & Biomedical Sciences, College of Veterinary Medicine, University of Minnesota, Twin Cities, MN, USA

**Keywords:** Dengue virus, dengue fever, Aedes aegypti, mosquitoes, vector-borne disease

Dengue virus (DENV) is the causative agent of dengue fever [1,2]. Dengue fever cases have increased significantly worldwide within the last two decades. A recent estimate indicates that as many as 390 million DENV infections occur each year, and that many infected individuals are asymptomatic or subclinical [3]. Only about one in four persons infected the first time develop symptoms, such as fever, headaches, fatigue, nausea, vomiting, a skin rash that looks like measles, and/or extremely painful body aches (and hence it’s sometimes being referred to as break-bone fever). Most inflicted people recover in 1–2 weeks, but about 1 in 20 people can develop more severe forms of the disease known as dengue hemorrhagic fever and dengue shock syndrome that can be fatal. The more times a person is infected with DENV, the higher the risk they are for severe and lethal complications. Dengue hemorrhagic fever has the highest case numbers of all known viral hemorrhagic fevers in the world, surpassing Ebola and Lassa fever cases, for example. As with all known viral hemorrhagic fever diseases, there is currently no specific drug to treat dengue fever, and supportive care (e.g. providing rest and fluids and acetaminophen as pain and fever reducer) is the only remedy.

DENV normally circulates in many tropical regions across the globe, but recent evidence suggests that local and imported dengue cases have been found in other regions of the world (Figure 1). So far this year (2024), dengue cases in South America have been reported earlier than expected and are in record high numbers. Latin America has seen its worst dengue outbreak on record so far this year, with case numbers in the first 4.5 months of 2024 already 238% higher than they were at this time last year, according to data from the Pan American Health Organization (https://www.paho.org/en/documents/situation-report-no-18-dengue-epidemiological-situation-region-americas-epidemiological). Dengue fever cases are 437% higher this year than the five-year average in Latin America. In recent years, the epidemic has spread to parts of southern Brazil and northern Argentina, where DENV wasn’t a serious issue in the past. According to the World Health Organization (WHO), Brazil, Argentina, and Paraguay have seen the highest number of dengue cases among 46 countries in the Americas so far this year (2024).

**Figure 1.**
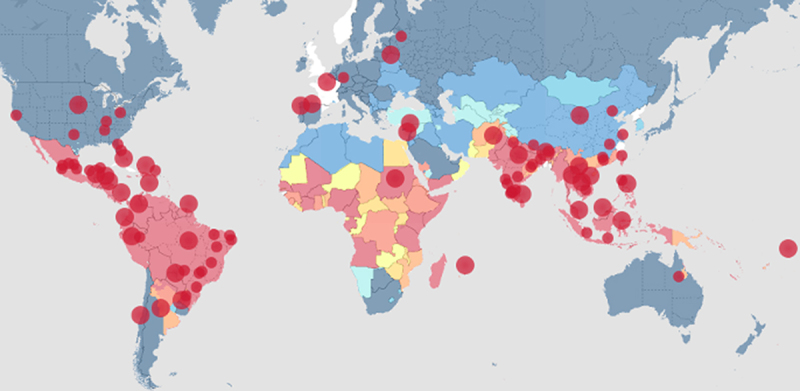
Recent (heatmap) reports of local or imported DENV cases from official, newspaper, and other media sources. Image and data were derived from an online source (heatmap.org/dengue/en/) as of 11 June 2024.

Puerto Rico, a place where dengue outbreaks have frequently happened, more than 400 people (>60% of patients) have been hospitalized in late March 2024 for dengue fever that have overwhelmed its healthcare system. As previously mentioned, cases of dengue fever have exceeded historical numbers in Puerto Rico and other Latin American countries thus far this year. Faced with a total of reported 7,861,445 suspected cases, resulting in a cumulative incidence of 833 per 100,000 population and accounting for 3.5 million cases and over 1,000 deaths due to dengue fever in Latin America, several countries in the region, including Brazil, Peru, Honduras, as well as the Commonwealth of Puerto Rico (an unincorporated island territory of the U.S.A. in the Caribbean Sea) have recently declared a state of emergency over dengue fever (https://www.nbcnews.com/news/latino/dengue-fever-outbreak-puerto-rico-creates-public-health-emergency-rcna145470). It’s worth noting that the last time that Puerto Rico declared a dengue fever epidemic was in 2012 when at least 199 people died from the infection [4]. The latest data collected by the Pan American Health Organization (PAHO) indicated that the entire South American region has already reported more than 8.65 million dengue fever cases in the first 5 months of 2024, which have shattered the recorded 4.5 million cases reported in 2023.

Whereas dengue outbreaks have historically occurred in the Americas every three to four years, it appears more frequently (i.e. annually) in recent years and in some unexpected places that have experienced unusually warm(er) weather that has created new mosquito’s habitats to allow the insect to breed all year long. The *Aedes aegypti* mosquito species that carries DENV is now endemic in the southern parts of the U.S.A., but more recently, it has also been found as far north as northern California (Bay area) and Washington, D.C. As a result, a recent study has predicted an additional 2 billion people, who will be at risk for dengue fever by 2080 [5]. The fact that *Aedes aegypti* mosquitoes are found in places outside their normal geographical range doesn’t always mean that they are carrying DENV, but recent epidemiological evidence suggests that they could serve as a natural carrier for the virus in certain places in the continental U.S.A. For example, Florida reported 176 cases of dengue fever earlier this year (2024) with a vast majority in people infected with DENV in other countries (e.g. Brazil or Cuba), and thus, those were referred to as travel-associated dengue cases. In contrast, the Florida Health Department has reported seven cases of locally transmitted DENV transmission in the state so far this year (https://www.floridahealth.gov/diseases-and-conditions/mosquito-borne-diseases/_documents/2024–20-arbovirus-surveillance.pdf). Locally transmitted dengue fever cases are defined as cases in which the persons have been infected while residing in the U.S.A. For example, in 2023, the Florida Health Department reported 173 locally transmitted dengue fever cases with most of them in Miami-Dade County. While locally transmitted dengue fever cases are still relatively rare in the continental U.S.A., they have recently been reported for the first time in some states besides Florida. For instance, last October, health officials reported the first case of locally transmitted dengue fever in southern California (Pasadena) (https://www.cityofpasadena.net/public-health/news-announcements/pasadena-reports-extremely-rare-case-of-locally-acquired-dengue-exposure-risk-to-local-residents-remains-very-low/). Local DENV transmissions have also been reported in Arizona and the southern coast of Texas.

The situation isn’t necessarily unique for North America as the *Aedes aegypti* mosquitoes have been found in 22 European countries and are thought to be responsible for locally transmitted dengue fever cases in France, Italy, and Spain. It is worth noting that dengue fever cases are known to occur in about 129 countries worldwide (Figure 1), with roughly half the world’s population at risk for DENV infections, according to the World Health Organization. More than 4.5 million cases of dengue fever had been reported as of November 2023, with more than 4,000 deaths due to dengue fever in 80 countries.

Development of dengue vaccines began in the 1920s, but the effort had been stymied by the need to create a protective immunity against all four DENV serotypes [6]. As of 2023, there are two commercially available vaccines, namely the CYD-TDV (brandname: Dengvaxia) and the TAK-003 or DENVax (brandname: Qdenga) dengue vaccines produced and sold by Sanofi Pasteur and Takeda, respectively. The CYD-TDV (Dengvaxia) vaccine is a recombinant live-attenuated tetravalent chimeric vaccine that is based on the attenuated 17D strain of the yellow fever virus to produce the PrM (pre-membrane) and E (envelope) structural proteins of the four DENV serotypes, and is administered as three separate injections, with the initial dose followed by two additional shots given six and twelve months later. It has been approved for use in 19 countries and the European Union (EU) (https://www.fda.gov/news-events/press-announcements/first-fda-approved-vaccine-prevention-dengue-disease-endemic-regions), but it is not FDA-approved for use in the U.S.A. and is not for individuals who have not been previously infected by any DENV serotype or for whom this information is not known, which means that many people are ineligible for the vaccine. This is due in part to the fact that while the vaccine has been shown to be partially effective in preventing DENV infection, it may lead to a higher risk of severe disease in those who have not been previously infected and then do go on to contract the disease. Likewise, the TAK-003 or DENVax (Qdenga) vaccine is a recombinant live-attenuated chimeric vaccine that is based on the DENV2 backbone to express DENV1, DENV3, and DENV4 components and is approved for use only in the EU in 2022 and in the United Kingdom, Brazil, Argentina, Indonesia, and Thailand in people who have not previously been infected with any DENV serotype, even though recent clinical data have indicated that this vaccine has demonstrated long-term efficacy and safety against all four DENV serotypes in previously DENV exposed individuals as well as against DENV-1 and DENV-2 infections in DENV-naive individuals [7,8]. In addition to those two commercially available dengue vaccines, there are other dengue vaccine candidates in development that include live attenuated, inactivated, DNA, and protein subunit vaccines [6].

Future efforts include further development of a safe and efficacious vaccine, such as the TAK-003 DENVax (Qdenga) vaccine or others, and antiviral drugs against DENV infections. In the meantime, it’s important for people who are traveling to places that are endemic for DENV to use insect-repellant and wear long sleeve shirts and pants to avoid mosquito bites, especially during the daytime, and to use bed nets during nighttime. In areas where it is hospitable for mosquitoes and mosquito breeding, people should either cover or empty standing water and make their yards less appealing for mosquito breeding. All efforts are necessary to curb the spread of DENV in endemic regions throughout the globe as well as in new areas in the Americas, EU, and elsewhere where DENV is becoming more commonly found to prevent future outbreaks and its pandemic potential.

## Data Availability

No primary data is included in this article.
